# Pacemaker therapy through a sex-specific lens: a narrative review of clinical and procedural disparities

**DOI:** 10.3389/fcvm.2026.1763436

**Published:** 2026-03-24

**Authors:** Ibrahim Antoun, Alkassem Alkhayer, Ahmed Abdelrazik, Mahmoud Eldesouky, Kaung Myat Thu, Mokhtar Ibrahim, Harshil Dhutia, G Andre Ng, Riyaz Somani

**Affiliations:** 1 Department of Cardiology, University Hospitals of Leicester NHS Trust, Glenfield Hospital, Leicester, United Kingdom; 2 Division of Cardiovascular Sciences, College of Life Sciences, University of Leicester, Leicester, United Kingdom; 3 Department of Cardiology, Guys and St Thomas’s Hospital, London, United Kingdom; 4 Department of Cardiology, Ain Shams University, Cairo, Egypt; 5 Department of Research, National Institute for Health Research Leicester Research Biomedical Centre, Leicester, United Kingdom; 6 Leicester British Heart Foundation Centre of Research Excellence, Glenfield Hospital, Leicester, United Kingdom

**Keywords:** cardiac defibrillator, gender disparity, implantable cardiac devices, pacemaker, sex disparity

## Abstract

Pacemaker therapy is a cornerstone in the management of bradyarrhythmias, yet accumulating data highlight important sex-based disparities across indications, procedural care and long-term outcomes. Women are consistently under-represented among pacemaker recipients, tend to be older at implantation, and are more frequently admitted via emergency pathways despite substantial symptom burden from sinus node dysfunction. Men more often receive pacemakers for atrioventricular conduction disease, at younger ages and with greater cumulative exposure to long-term pacing and device-related procedures. Procedurally, women have higher rates of acute complications, particularly pneumothorax, pocket haematoma and lead perforation, reflecting differences in body size, venous anatomy and myocardial vulnerability. At the same time, men appear to carry a higher long-term risk of device infection. Historical inequities in device selection, with lower use of dual-chamber systems in women, are narrowing but remain a concern in some cohorts. Long-term outcomes suggest that although women experience more early procedural risk, their survival after pacemaker implantation is at least comparable to, and sometimes better than, that of men, with distinct profiles of heart failure, pacing-induced cardiomyopathy and quality-of-life trajectories. Emerging technologies such as leadless pacemakers and conduction system pacing may mitigate several of these disparities by avoiding leads and pockets, reducing dyssynchrony and lowering complication rates in high-risk subgroups. However, women remain under-enrolled in device trials, mechanistic explanations for many sex differences are incomplete, and systematic sex-specific reporting is often lacking. Addressing these gaps is essential to deliver equitable, evidence-based and personalised pacemaker therapy for both women and men. However, most available evidence derives from high-income countries and selected national registries, with limited representation from low and middle-income regions, which may constrain the generalisability of these findings across diverse healthcare settings.

## Introduction

Pacemaker therapy is a mainstay treatment for bradyarrhythmias, significantly improving survival and quality of life in patients with symptomatic sinus node dysfunction or atrioventricular (AV) conduction block. However, growing evidence indicates there are important sex-based differences in how patients are selected for pacemaker implantation, the types of devices they receive, and the outcomes they experience. Historically, cardiovascular care has shown disparities, with women often receiving interventions less frequently or later than men in comparable conditions. Pacemaker utilisation is no exception: contemporary data from large cohorts demonstrate that men not only receive pacemakers more frequently but also at an earlier age, on average, than women (1). This narrative review examines the current evidence on sex differences in pacemaker therapy, covering indications and referral patterns, procedural characteristics, complications, and long-term outcomes. It discusses the clinical implications of these disparities.

Women and men with bradyarrhythmias can present with differing clinical profiles, necessitating a sex-specific lens in evaluating pacemaker therapy (2). We will explore how indications for pacing may vary by sex and whether referral pathways introduce bias or delays. We then discuss procedural aspects, including device selection (single vs. dual-chamber systems) and technique, and highlight any distinctive considerations for female vs. male patients. Differences in complication rates are reviewed in light of recent evidence. We also compare survival, rehospitalisation, and quality-of-life outcomes after pacemaker implantation in women vs. men. Finally, we consider newer modalities, such as leadless pacemakers and conduction system pacing, to determine whether they offer advantages or pose challenges in both sexes, and to identify knowledge gaps and future research priorities to ensure pacemaker therapy is optimised for all patients.

## Sex differences in indications and referral pathways

Multiple studies have documented that the underlying indications for pacemaker implantation differ between men and women. Women more frequently require pacemakers for sick sinus syndrome (SSS) or sinus node dysfunction, whereas men have a higher prevalence of AV block or His–Purkinje system disease leading to complete heart block (3). For example, in a large registry from China, over 60% of female pacemaker patients had SSS as the primary indication, significantly more than in males, who more often presented with AV block (3). Similarly, a study comparing left bundle branch area pacing vs. conventional pacing found that complete AV block was the indication in about 50.7% of male patients but in only 41.7% of female patients. In contrast, sinus node dysfunction was the indication in 57% of women (vs. 48% of men) (4). These findings suggest potential sex-related differences in bradyarrhythmia patterns, although the underlying mechanisms remain incompletely understood. Observational data from pacemaker cohorts suggest that women more frequently undergo implantation for sinus node dysfunction, whereas men more often receive pacemakers for atrioventricular conduction disease. However, these findings reflect treated populations and may be influenced by referral patterns, clinical decision-making, and access to care, rather than true sex-specific differences in disease incidence or prevalence (5). Accordingly, these differences should be interpreted as patterns of device utilisation rather than definitive evidence of biological predisposition.

Referral pathways and timing may also differ by sex. Population-based data from Australia (2009–2018) found that men had a higher pacemaker implantation rate, and this gap widened with age. Beyond age 50, pacemaker implantation rates in men are approximately twice those in women of the same age (1). However, implantation rates reflect healthcare utilisation and clinical decision-making rather than the true incidence or prevalence of bradyarrhythmias, which may differ and are not consistently reported in a sex-stratified manner. Therefore, differences in implantation rates should not be interpreted as direct evidence of sex-specific disease risk, but rather as a combination of epidemiology, referral patterns, and treatment thresholds.

Importantly, women were more often admitted via emergency or urgent pathways than via elective referrals. Consistently, an analysis of a large device database noted that women were more likely to require an emergency pacemaker implantation than men. These patterns raise concern that bradyarrhythmias in women might be under-recognised until more acute, or that healthcare providers might be slower to refer women for permanent pacing until symptoms become severe (6). Societal biases and differences in how men and women report symptoms could play a role. Ensuring that referral for pacemaker evaluation is based on objective criteria and not influenced by patient sex is a key step toward equity. Increased awareness that women can and do suffer high-grade AV block or severe sinus node dysfunction (even if at slightly older ages or different etiologies) may help close the referral gap (7). Global generalisability remains limited by uneven availability of national device registries and inconsistent sex-stratified reporting, particularly in low- and middle-income countries. Where data exist from resource-constrained settings, indications and outcomes appear to be shaped not only by biological sex and comorbidity, but also by access to elective referral, device affordability, and follow-up capacity. Single-centre and regional reports from Sub-Saharan Africa and East Africa describe substantial burdens of lead dislodgement and pocket infection, as well as challenges related to delayed presentation and limited post-implant surveillance (8, 9). These contextual factors may amplify complications that are less common in high-income registries and may alter observed sex patterns if women face additional barriers to referral and elective implantation.

## Procedural characteristics and device selection

Once the decision to implant a pacemaker is made, device selection and procedural approach can also vary by sex. One historical observation is that women have been less likely than men to receive dual-chamber pacemakers (which pace both the atrium and the ventricle) in certain contexts, even when indications for dual-chamber pacing would otherwise be met. An analysis of an early-2000s German quality-control program found that, among patients aged ≥80 years, men were significantly more likely than women to receive dual-chamber devices for the same indications (AV block or sick sinus syndrome) (10). Overall, that study reported sex as an independent determinant of pacemaker type, with women receiving approximately 20% fewer dual-chamber or rate-responsive systems than men, possibly reflecting a combination of clinical factors (e.g., more women had chronic atrial fibrillation and thus were only warranted single-chamber ventricular pacemakers) and potential biases. In more recent practice, however, these differences have narrowed (11).

Notably, apparent discrepancies between earlier European datasets and more contemporary Asian registries highlight the importance of contextual interpretation. The German quality-control programme, conducted in the early 2000s, reflected practice patterns at a time when device selection was influenced by evolving guideline adoption, more limited device miniaturisation, and greater variability in operator preference. In that setting, women were less likely to receive dual-chamber systems, even after accounting for pacing indication. In contrast, more recent registry data from China indicate comparable or higher use of dual-chamber devices among women, likely reflecting changes in guideline implementation, broader availability of dual-chamber systems, and differences in case mix, particularly the higher prevalence of sinus node dysfunction among female recipients.

These differences are also influenced by population characteristics. Women undergoing implantation are typically older and more likely to have atrial fibrillation, which may appropriately favour single-chamber ventricular pacing in some cases. However, these clinical factors do not fully account for the observed disparities. Historical underutilisation of dual-chamber devices in women may also reflect referral and treatment biases, including under-recognition of symptoms, later presentation, and more conservative device selection in older or smaller-bodied patients. In addition, healthcare system factors, such as reimbursement policies, device costs, and access to electrophysiology expertise, may influence device choice across regions.

Taken together, the available evidence suggests that sex differences in device selection are not solely biologically determined but arise from an interplay between clinical indication, patient characteristics, and healthcare delivery factors. Contemporary data indicate that when these factors are appropriately accounted for, women are as likely as men to receive dual-chamber systems, supporting the importance of indication-driven rather than sex-driven decision-making (12).

Beyond chamber configuration, procedural techniques and technical considerations may differ subtly for female patients. Women generally have a smaller body size and vein diameter, which can pose challenges during lead implantation. Operators must take care with venous access (subclavian or axillary vein puncture), as smaller veins may increase the difficulty of lead advancement or raise the risk of long-term venous occlusion (13). Some implanters report using the cephalic vein cut-down approach more often in women to avoid complications associated with subclavian venous access (though data on this practice are anecdotal) (14). Additionally, women's thinner pectoral fat pads and finer skin can make the pacemaker pulse generator more prominent and the pocket creation more delicate, with implications for cosmesis and comfort (15). Device miniaturisation over the past few years (smaller, thinner pacemaker can sizes) has improved comfort for patients with smaller body habitus. Yet women remain more likely to report device visibility, local discomfort, or irritation due to pressure from clothing over the implantation site (16). Post-implant. Procedurally, careful pocket placement and closure are especially important in female patients to reduce the risk of erosions or chronic pain from device rubbing (17).

Notably, there are also electrophysiologic differences during implantation. Women tend to have higher pacing thresholds and lower intrinsic amplitudes, at least in the atrium, at implant than men (10). These differences might reflect smaller atrial size or differences in atrial myocardial characteristics, underscoring the importance of meticulous lead positioning and threshold testing in women (2). Fortunately, modern pacemakers can automatically adjust sensitivity and output. Hence, the clinical impact of these sex differences in electrical parameters is minimal as long as they are recognised (e.g., ensuring a safety margin for capture in women who may have slightly higher thresholds).

Regarding the adoption of device technologies, there is evidence of disparities. In some cohorts, men were more often implanted with remote-monitoring-enabled devices than women (18). However, this gap has likely narrowed with the ubiquitous availability of remote follow-up across manufacturers. The reasons for any such disparity could include historical differences in healthcare access or in providers' perceptions of patients' ability to use remote technology. There is no inherent reason women should receive older device models; therefore, efforts should be made to ensure equal access to the latest pacemaker technologies (e.g., MRI-safe devices and remote monitoring) for all patients (19).

## Complications

One of the clearest disparities in pacemaker therapy is seen in acute complication rates, which tend to be higher in women than in men (13). Large real-world analyses have confirmed that women experience more procedure-related complications during or shortly after pacemaker implantation (1). In a statewide Australian cohort of over 28,000 pacemaker procedures, women had an in-hospital complication rate of 8.2% vs. 6.6% in men (*p* < 0.001) (1). After adjusting for other factors, male sex was associated with significantly *lower* odds of complications (adjusted OR ∼0.79), implying female sex conferred about 25% higher odds of an adverse event during the implant hospitalisation. Similarly, an earlier multicenter study in Europe found total acute complication rates of 5.8% in women vs. 4.7% in men, with women having a roughly 30% higher risk (OR ∼1.3) (10). The kinds of complications driving this disparity appear to be those related to the procedure and access. Notably, women have significantly higher rates of pneumothorax and pocket hematoma formation after pacemaker surgery (20). Pocket hematomas (significant bruising or bleeding in the device pocket) are also reported more frequently in female patients, which may be related to tissue fragility or a smaller pocket size relative to device size (20). These hematomas can be more than cosmetic; if severe, they increase the risk of infection and may prolong hospital stay. Careful haemostasis and, perhaps, closer postoperative monitoring in women (especially those on anticoagulation) are sensible precautions. However, other studies have reported similar adverse events in men and women with dual-chamber pacemakers (21). Although consistent sex differences in complication rates are observed, the underlying mechanisms remain incompletely defined, and most explanations are derived from observational data rather than direct mechanistic studies.

Cardiac perforation is an uncommon but serious complication of pacemaker implantation, with an incidence typically below 1% (22). Female sex has been identified as an independent predictor of lead perforation in several observational studies. In a large single-centre analysis of over 3,800 active-fixation leads, female sex was associated with a more than threefold increased risk of clinically significant perforation (OR 3.14, 95% CI 1.07–9.22) (23). Advanced age and right ventricular apical lead positioning were also identified as key risk factors (23). The increased susceptibility in women may relate to smaller cardiac dimensions and thinner myocardial walls, although mechanistic data remain limited (13). When perforation occurs, clinical presentation ranges from asymptomatic lead migration to pericardial effusion and cardiac tamponade requiring urgent intervention. Most cases can be managed with pericardiocentesis and lead repositioning, with surgical intervention rarely required in contemporary practice (24). These observations are consistent across large registry and cohort studies of device-related complications. Procedural strategies to mitigate perforation risk include careful lead positioning, avoidance of excessive lead slack or force during fixation, and consideration of non-apical right ventricular pacing sites where feasible (24). While these approaches are widely adopted in clinical practice, supporting evidence is largely observational, and sex-specific procedural strategies require further study. Findings from large observational cohorts are supported by systematic reviews and meta-analyses conducted across multiple regions. A comprehensive meta-analysis of cardiac implantable electronic device infections identified consistent risk factors across diverse populations, including device complexity, repeat procedures, and comorbidity burden (25). Similarly, systematic reviews and meta-analyses examining lead-related complications demonstrate variability in thrombotic and venous outcomes across patient populations and healthcare settings (26). These findings highlight the influence of both patient-level and system-level factors on the risk of complications. However, sex-stratified analyses within these meta-analyses remain limited, and the consistency of sex-based differences across global settings is not well established. This underscores the need for future multinational meta-analyses with prespecified sex-specific reporting.

Regarding vascular access-site complications, women may again have a slightly different profile. Pneumothorax has been reported more frequently in women, and this may relate to anatomical differences such as smaller venous calibre and proximity to the lung apex, although direct mechanistic evidence remains limited (10). Arterial puncture or bleeding at the access site is not commonly stratified by sex in the literature, but smaller vessel size could intuitively make subclavian vein entry more delicate (27). Pacemaker leads are usually placed via the subclavian or axillary vein; if the subclavian stick is high, there's a risk of hitting the subclavian artery or lung apex. Meticulous technique is therefore important for all patients, and especially in women, where the margin for error is small. Sex-stratified data on venous thromboembolism after pacemaker implantation remain limited and are not consistently reported. Available studies describe thromboembolic events and lead-related thrombosis after implantation, but do not provide robust, consistent evidence for a clear sex difference in risk (26, 28, 29).

Infections in the acute post-operative period (pocket infections or endocarditis presenting during the implant hospitalisation) are relatively rare and not strongly differentiated by sex in acute datasets; the overall infection rate at initial implant is usually <1%–2% (30). If anything, Several cohorts and risk factor analyses report a male predominance among CIED infection cases and, in some analyses, male sex remains associated with infection after adjustment for device type and other clinical factors (25, 31). Still, those infections typically occur later in the course. The early pocket infections that do occur may not exhibit a clear sex predilection after adjustment.

Beyond the acute phase, pacemaker patients face potential long-term complications involving the leads and device, and here, too, some sex-specific patterns have been observed (2). One long-term issue is lead durability and malfunctions (like lead fracture, insulation failure, or chronic lead dislodgement). Most large series have not found a stark sex difference in lead longevity or the need for lead reinterventions, after accounting for the fact that women live longer on average (and thus have leads in place for more years) (32). In the Vienna cohort study with up to 10-year follow-up, the incidence of lead replacement or lead revision was similar between women and men (2). This suggests that once a pacemaker is in place, women do not necessarily experience more lead failures than men, a reassuring finding that modern leads perform equally well across patient sexes. However, women's smaller vein diameters may predispose them to chronic subclavian vein stenosis or occlusion associated with indwelling leads (33). While not always symptomatic, such venous occlusion can complicate future lead additions or revisions. The incidence of significant venous occlusion on imaging can be 20%–30% in CIED patients overall; although data by sex are sparse, one might expect higher relative rates in women (given smaller initial vein calibre) (34). This remains an area for further research. Clinicians should maintain a high index of suspicion for upper-extremity swelling or venous congestion in any pacemaker patient and plan to manage the cumulative lead burden (e.g., by considering laser extraction of old leads if new ones are needed). Emerging data from low- and middle-income countries (LMIC) suggest that sex-based disparities in procedural risk are also present, and may be more pronounced, in resource-limited environments. In an LMIC cohort, female sex was significantly associated with higher rates of pacemaker-related complications (*p* = 0.04) (8). Similarly, a recent study by Nasir et al. reported a 4.5-fold increased risk of procedural complications in women compared with men (35). These findings are directionally consistent with observations from high-income registries but may reflect additional contributing factors, including delayed presentation, differences in access to elective implantation, variability in operator experience, and constraints in peri-procedural care and follow-up infrastructure. Together, these data suggest that sex disparities in complication risk are likely influenced by both biological factors and healthcare system characteristics and may be amplified in LMIC settings.

Infection is another long-term complication of concern. After the initial 90-day post-implant period, devices can become infected years later, usually via hematogenous seeding or erosion (31). Several studies have suggested that male sex is a risk factor for CIED infections, partly because males more often have risk factors like prior device interventions, ICD/CRT devices, or comorbid conditions that predispose to infection (36, 37). However, crude sex distributions among infected cases cannot be interpreted as sex-specific risk because overall device implantation rates are higher in men. Where sex-specific risk is evaluated, conclusions should be based on incidence rates or adjusted analyses rather than case proportions (25, 38). On the other hand, women who do develop device infections might fare worse; one study noted that female patients had significantly higher mortality associated with CIED infections compared to males (39). This could be due to women being older at the time of implantation or to infections being recognised later. Regardless, infection prevention (through sterile technique, antibiotic prophylaxis, and, where appropriate, adjuncts such as antibacterial envelopes or barrier drapes) should be optimised for all patients. Given the potentially worse outcomes in women with device infection, prompt diagnosis and aggressive management (including complete hardware removal) is especially crucial in female patients who present with signs of infection (40).

Tricuspid valve dysfunction is a notable long-term complication specifically tied to transvenous leads. The presence of a lead crossing the tricuspid valve can cause or exacerbate tricuspid regurgitation (TR) over time, due to mechanical impingement or fibrosis on the valve apparatus (41). Significant TR can lead to right heart failure symptoms and a worse prognosis. Studies have shown that new or worsened TR after pacemaker implantation is not uncommon and is associated with increased mortality in the long run (2). While both men and women can develop lead-related TR, some evidence suggests women might be *somewhat* more susceptible to its hemodynamic effects, as Women may experience different haemodynamic consequences of lead-associated tricuspid regurgitation, although sex-specific data on tolerance and clinical impact are limited (42). However, concrete data stratifying TR incidence by sex are limited. New pacing strategies, such as lead placement on the septal side of the tricuspid valve (to avoid the free wall) or the use of leadless pacemakers, can circumvent this issue; further details are provided in a later section. Associations between sex and long-term outcomes should be interpreted cautiously, as available data are largely observational and may be influenced by baseline differences in age, comorbidity burden, and pacing indications.

When long-term pacemaker system issues arise, such as a lead requiring extraction due to infection or malfunction, sex differences in extraction outcomes have been reported. A recent retrospective analysis of transvenous lead extraction (TLE) procedures found that women had a higher incidence of minor complications during extraction (17.2% vs. 2.5% in men) (43). These were minor issues (e.g., transient arrhythmias and minor bleeding) and did not translate into differences in major complication or procedural success rates, which were excellent in both sexes. The higher minor complication rate could reflect the greater technical challenge posed by smaller vasculature or more fibrotic leads in women (who were, on average, older at implantation and had longer-implanted leads). It emphasises that extraction in female patients should be performed with meticulous technique, often at experienced, high-volume centres, to ensure safety. Importantly, long-term outcomes after extraction (freedom from reinfection or survival) did not differ by sex (43). Once the immediate procedure is completed, women benefit as much as men from the successful removal of infected or malfunctioning leads (40).

An additional long-term consideration is pacing system upgrades, for instance, a patient with a longstanding single-chamber pacemaker who later develops a need for CRT due to heart failure. Some data indicate that women with pacemakers for AV block were less likely to develop pacing-induced cardiomyopathy or need an upgrade to CRT compared to men (44). In an analysis of patients undergoing CRT upgrades, women paced for AV block were older but had fewer comorbidities and significantly less new-onset heart failure than their male counterparts (44). This suggests that women may tolerate long-term RV pacing better, possibly because they more often have preserved left ventricular function and fewer ischemic heart disease issues at baseline when they receive a pacemaker. Men, especially younger men with AV block due to infarction or other pathology, might be more prone to the deleterious effects of chronic RV apical pacing (dyssynchrony leading to heart failure) (45). If confirmed, this could mean that, in men, we should be more proactive in implementing programming strategies to minimise RV pacing or consider conduction system pacing (to be discussed) to avert cardiomyopathy. Meanwhile, ensuring women are appropriately monitored for any signs of pacing-related dysfunction remains important, even if their risk is statistically lower (45).

QoL over the long term with a pacemaker is generally high for both sexes, but some differences have been noted in patient-reported outcomes (21). Pacemakers relieve disabling symptoms like syncope and fatigue from bradycardia, which results in improved functional status and well-being. However, psychosocial and physical adjustments to having an implanted device might differ between men and women. In some surveys, male patients reported better physical functioning and less role limitation due to emotional or dyspnea symptoms after pacemaker implantation compared to female patients (46). Women may report lower scores in physical QoL domains, potentially because they are older, on average, at implantation and have more age-related functional limitations, or because they have greater concerns about the device (e.g., body image, anxiety) (15).

On the other hand, improvements in arrhythmia-related symptoms are observed in both sexes. It's worth noting that dual-chamber pacing tends to confer better exercise capacity and QoL than single-chamber ventricular pacing, by preserving AV synchrony (2). This is relevant given the higher prevalence of single-chamber pacemakers in men with permanent AF. Those patients (often male) might have inherently lower QoL due to their underlying condition rather than the pacemaker itself. Ensuring women receive dual-chamber devices when indicated (since they benefit from the associated QoL advantage) is important. Overall, ongoing support and education post-implant can help all patients, and particularly women who may have more questions or anxiety, to adapt to living with a pacemaker and fully trust the device. Studies have shown that targeted education and follow-up can significantly improve pacemaker acceptance and patient satisfaction among women, underscoring the role of patient-centred care in addressing outcome gaps (21).

## Sex-specific considerations in leadless pacing and conduction system pacing

Early leadless pacemaker platforms were predominantly single-chamber right ventricular systems (47). Historically, this has limited their suitability for patients who require consistent atrioventricular synchrony, including many individuals with sinus node dysfunction who would otherwise benefit from atrial-based or dual-chamber pacing. This is not a sex-specific limitation. It reflects pacing indication and rhythm status. Because sinus node dysfunction is more common among female recipients in several cohorts, women may be less frequently eligible for single-chamber ventricular leadless pacing when AV synchrony is a priority. However, women are not inherently suboptimal candidates for leadless pacing (2). When the clinical indication matches a single-chamber ventricular strategy, outcomes in women and men appear comparable in contemporary registry analyses.

This framework also reconciles differences observed across transvenous device selection studies. Historical underuse of dual-chamber transvenous systems in women likely reflected a combination of era effects, local practice patterns, and potential bias. In contrast, higher contemporary dual-chamber implantation rates in women in some registries align with indication-driven selection in sinus node dysfunction. Leadless pacing should similarly follow indication-based principles. The expansion of leadless technologies, including AV-synchrony capable systems and dual-chamber leadless pacing, may reduce prior constraints related to AV synchrony and could broaden eligibility for both women and men (20). Real-world data on leadless pacemaker (LPM) implants by sex have started to emerge. A recent multicenter registry (iLEAPER) analysis reported that women were underrepresented among leadless pacemaker recipients; only ∼35% of LPM patients were female (48). Early leadless pacemaker systems were limited to single-chamber right-ventricular devices, such as the Micra platform; however, dual-chamber leadless systems have since been developed and are now available, thereby expanding the potential indications for leadless pacing (47). Historically, the availability of only single-chamber leadless systems limited their use in patients requiring atrioventricular synchrony, including many with sinus node dysfunction. With the emergence of dual-chamber leadless technologies, this limitation may be reduced, although real-world sex-specific data remain limited. Nonetheless, among women who received leadless pacemakers, outcomes have been excellent. The iLEAPER study found no significant sex differences in major complication rates or all-cause mortality after leadless pacemaker implantation (48). In propensity-matched comparisons, female sex was not associated with an increase in LPM-related complications (hazard ratio ∼2.0, *p* = 0.19, not statistically significant), and mortality rates were virtually identical between women and men. The electrical performance of the leadless devices was also on par. The only notable difference was that women had slightly higher pacing impedance measurements at both implant and follow-up (at 2 years, median impedance ∼670 Ω in women vs. 616 Ω in men) (48). This higher impedance in females likely stems from their smaller cardiac chamber size (leading to less current spread). Still, importantly, it remained within normal limits and did *not* adversely affect pacing efficacy. The overall message is that leadless pacemaker therapy is equally safe and effective in women, and the underutilization in women is more a function of referral patterns and device selection criteria than of performance. As leadless technology expands (e.g., dual-chamber leadless systems in development), it will be crucial to enrol adequate numbers of female patients in trials to confirm their benefits. Leadless pacing could, in fact, eliminate some disparities, for instance, by avoiding pocket hematomas and lead perforations, which we know women are more prone to with transvenous systems. Leadless pacemakers demonstrated excellent outcomes and a low complication profile in both sexes (49). Future studies should examine whether adopting leadless devices reduces the sex-based complication gap. Current evidence is derived from registry and observational data, and sex-specific differences in outcomes require confirmation in prospective studies with balanced representation. Leadless pacing has been adopted unevenly across regions due to cost, reimbursement, and operator and cath lab infrastructure. Therefore, conclusions about leadless pacing as a disparity-mitigating strategy are most applicable to settings where leadless systems are accessible and supported by structured follow-up. In a large real-world registry analysis, women were underrepresented among leadless recipients, while major complications and device performance were comparable between sexes.

Conduction system pacing (CSP) refers to newer techniques such as His-bundle pacing (HBP) or left bundle branch area pacing (LBBAP), in which the pacemaker lead is placed in or near the native cardiac conduction system rather than the right ventricular apex. This approach can achieve more physiological ventricular activation and is used both for bradycardia (as an alternative to RV pacing) and for cardiac resynchronisation in heart failure (50). Given the known sex differences in response to traditional CRT, with women often deriving greater benefit from CRT than men, it is pertinent to ask if sex differences exist in response to CSP (51). Early evidence suggests there may be sex-specific outcomes with CSP as well. A large observational study comparing LBBAP to conventional biventricular pacing (BVP) for CRT found that female patients experienced significantly greater benefit from LBBAP than male patients (52). In that study, women had a 36% reduction in the combined endpoint of death or heart failure hospitalisation with LBBAP compared to BVP, whereas men did not see as large a difference. In other words, recruiting the native conduction system conferred a substantial advantage in women, who already tend to respond well to CRT but appeared to respond even better to left bundle pacing. The reasons are not entirely clear, but it could be related to women having smaller hearts and narrower baseline QRS durations. Thus, they achieve near-normal electrical synchrony with CSP, resulting in improved outcomes. Indeed, another analysis noted that differences in cardiac size and baseline QRS contributed partially to the sex-based variations in CSP outcomes (51). Female hearts, often less remodelled in non-ischemic cardiomyopathy, might normalise function more completely with physiological pacing (53).

When it comes to using CSP for basic bradycardia indications (as a substitute for standard RV pacing to prevent pacing-induced cardiomyopathy), both sexes appear to benefit from the improved synchrony. There is no strong evidence of a sex difference in the feasibility or acute success of His-bundle or left bundle lead implantation; success rates are high (>90%) in experienced hands for both women and men (51). Women's smaller septal size may make positioning slightly more challenging in theory; however, catheter techniques have been effective across body sizes. It is important, however, to ensure that women are not excluded from adopting these new techniques (54). If there is a bias wherein complex new procedures are offered more to male patients, that could exacerbate disparities. On the contrary, CSP might be especially useful for women with a long life expectancy and many years of pacing ahead. Early detection of the His-Purkinje system could prevent the decline in ventricular function that sometimes disproportionately affects men with decades of RV pacing.

Another aspect of CSP is the impact on tricuspid regurgitation: His-bundle pacing still uses a transvalvular lead (which can cause TR similarly to a conventional RV lead), but left bundle branch area pacing often involves septal lead placement that may avoid impinging the tricuspid leaflet (55). This could be beneficial in women, for whom severe TR is particularly poorly tolerated. As mentioned, leadless pacing avoids interaction with the tricuspid valve entirely. Thus, women with prior lead-related TR or at high risk of it might be good candidates for either CSP (if CRT is needed) or leadless RV pacing (if only backup pacing is required). Reported sex-related differences in response to conduction system pacing are hypothesis-generating and warrant validation in randomised and adequately powered studies.

## Artificial intelligence and sex-specific insights in pacemaker therapy

Artificial intelligence is increasingly being integrated into cardiac rhythm management and offers potential to address sex-based disparities in pacemaker therapy (56–60). Machine learning models applied to electrocardiography can improve detection of bradyarrhythmias, including sinus node dysfunction and conduction disease, and may enable earlier and more objective referral for pacing (61). This is particularly relevant for women, in whom symptoms may be under-recognised or attributed to non-cardiac causes. AI-enabled ECG analysis has also shown promise in identifying subclinical conduction abnormalities and predicting progression to high-grade atrioventricular block, thereby reducing delays in diagnosis and intervention (61).

Beyond diagnosis, AI-driven risk stratification tools may support procedural planning and the prediction of complications. Models incorporating clinical, imaging, and procedural variables have demonstrated the potential to predict risks such as lead perforation, pneumothorax, and device infection. Given the higher procedural risk observed in women, such tools could facilitate personalised procedural strategies, including access site selection, lead positioning, and closer post-procedural monitoring.

Artificial intelligence is also being explored in device management, including optimisation of pacing parameters, remote monitoring, and early detection of device-related complications (62). Automated algorithms can analyse large volumes of device data to identify patterns suggestive of lead dysfunction, arrhythmia burden, or deterioration in heart failure (40). These approaches may improve long-term outcomes and reduce healthcare utilisation in both sexes.

However, important limitations remain. Many AI models are trained on datasets that underrepresent women, raising concerns about bias and generalisability. Sex-specific performance of these models is rarely reported, and external validation in diverse populations is limited (63). Ensuring balanced datasets and transparent reporting of sex-stratified performance metrics is essential to avoid perpetuating existing disparities.

Future research should focus on integrating AI into clinical decision pathways while ensuring equitable performance across sexes. If appropriately developed and validated, artificial intelligence could play a key role in standardising referral, reducing procedural risk, and enabling more personalised pacemaker therapy.

## Knowledge gaps and future research priorities

Much of the current evidence describing sex differences in pacemaker therapy is observational, with limited mechanistic insight and inconsistent sex-stratified reporting across studies. This limits the ability to draw causal inferences and may contribute to residual bias in interpretation. Despite the insights gained from recent studies, significant knowledge gaps remain regarding sex disparities in pacemaker therapy. First, women are under-enrolled in many cardiac device trials and registries. Historically, female patients have comprised a minority of participants in pivotal device studies, leading to persistent uncertainties about how guideline recommendations apply by sex. One priority is to increase the representation of women in prospective pacemaker research, including trials of new pacing technologies, long-term outcome studies, and device safety monitoring. Ensuring sex-specific data reporting (beyond just adjusting for sex in multivariable models) will help clinicians understand if and why certain outcomes differ. For instance, if a new lead design dramatically reduces perforation in all patients, does it eliminate the female excess risk, or do women still have higher perforation even with the new lead? Such questions can only be answered if studies are adequately powered to compare subgroups.

Another area needing exploration is the pathophysiological basis of sex differences in pacing indications and complications. We know the what (e.g., women have more SSS, more pocket bleeds; men have more AV block, more infections), but not fully the why. Future research could investigate whether hormonal or genetic factors influence the development of conduction system disease differently in men vs. women. For example, does estrogen exposure protect against AV node fibrosis (hence fewer younger women with AV block) or, conversely, contribute to sinus node fibrosis? Are there differences in collagen deposition or ion channel function that predispose one sex to certain bradyarrhythmias? Similarly, why do women experience more pocket hematomas? Is it related to body fat distribution, differences in coagulation, or simply a smaller size relative to the device, which would lead to more bleeding? Understanding these mechanisms could inspire targeted strategies, such as refining surgical technique or implementing prophylactic measures (some centres use compressive dressings or longer observation for female patients' post-implant, but this is not yet evidence-based). A major limitation of the current literature is the scarcity of sex-stratified pacemaker data from South America, Africa, the Middle East, and several parts of Asia. Even when implantation data are available, definitions of complications, follow-up durations, and risk adjustment are heterogeneous, limiting cross-country comparisons. Future registries should prespecify sex-stratified analyses, report access pathway metrics (elective vs. emergency implantation), and capture setting-level determinants such as centre volume, access technique, device type availability, and post-implant follow-up infrastructure.

Device programming and management strategies tailored to sex is another fertile ground for research. Given that women, on average, have higher atrial pacing thresholds and possibly a greater tendency to chronotropic incompetence with age, should pacemaker programming (e.g., rate-response algorithms) be adjusted or optimised differently for women? Are there sex-specific differences in ideal AV delay or other programmable parameters that could improve outcomes? Thus far, guidelines do not distinguish programming by sex, but subtle differences in electrophysiology might merit investigation. For instance, the optimal settings for rate-adaptive pacing might differ if women generally have smaller stroke volume and might benefit from more aggressive rate response to activity, a hypothesis that could be tested.

From a clinical outcomes standpoint, a key question is how to eliminate the gap in complication rates. Future quality improvement initiatives could focus on reducing pneumothorax and hematoma in women, possibly through routine ultrasound-guided venous access (which is increasingly recommended for all patients), careful postoperative anticoagulation management, and the use of smaller-profile leads and sheaths. Device manufacturers might even consider developing *sex-specific device offerings* (for example, a slightly smaller pacemaker or a lead optimised for smaller vasculature). However, many current pacemaker models are already quite small; further innovation could benefit patients of small stature (disproportionately female). The concept of personalised medicine could extend to device therapy: perhaps one day a clinician will decide between a traditional and a leadless pacemaker, or between RV vs. conduction system pacing, partly based on the patient's sex, in combination with anatomy and clinical factors, to maximise efficacy and minimise risk.

Another research priority is the impact of pacemaker therapy on long-term outcomes like heart failure and AF, stratified by sex (64). We touched on evidence that men might have higher rates of pacing-induced cardiomyopathy and subsequent heart failure than women (44). This needs confirmation in broader populations. If true, it suggests that men might benefit from earlier adoption of alternatives to apical RV pacing (e.g., CSP) or from stricter programming to minimise the RV pacing percentage. Conversely, does long-term pacing increase atrial fibrillation risk differently in women vs. men? Some earlier studies indicated that dual-chamber pacing could reduce AF incidence overall compared with ventricular pacing, but whether one sex derives greater anti-arrhythmic benefit is unclear. Given that women have a higher baseline risk of stroke from AF (65), understanding any pacing-related promotion or prevention of AF in either sex is worthwhile.

The psychosocial aspect is another gap. Women's QoL and psychological adjustment after pacemaker implantation might benefit from targeted interventions. Research could explore if women experience more anxiety or depression related to having a device (some ICD literature suggests women do experience more anxiety with implants). If so, tailored counselling or support groups for female CIED patients could be developed. Patient-reported outcome measures should be included in pacemaker follow-up studies to capture potential differences in needs.

**Figure 1:**
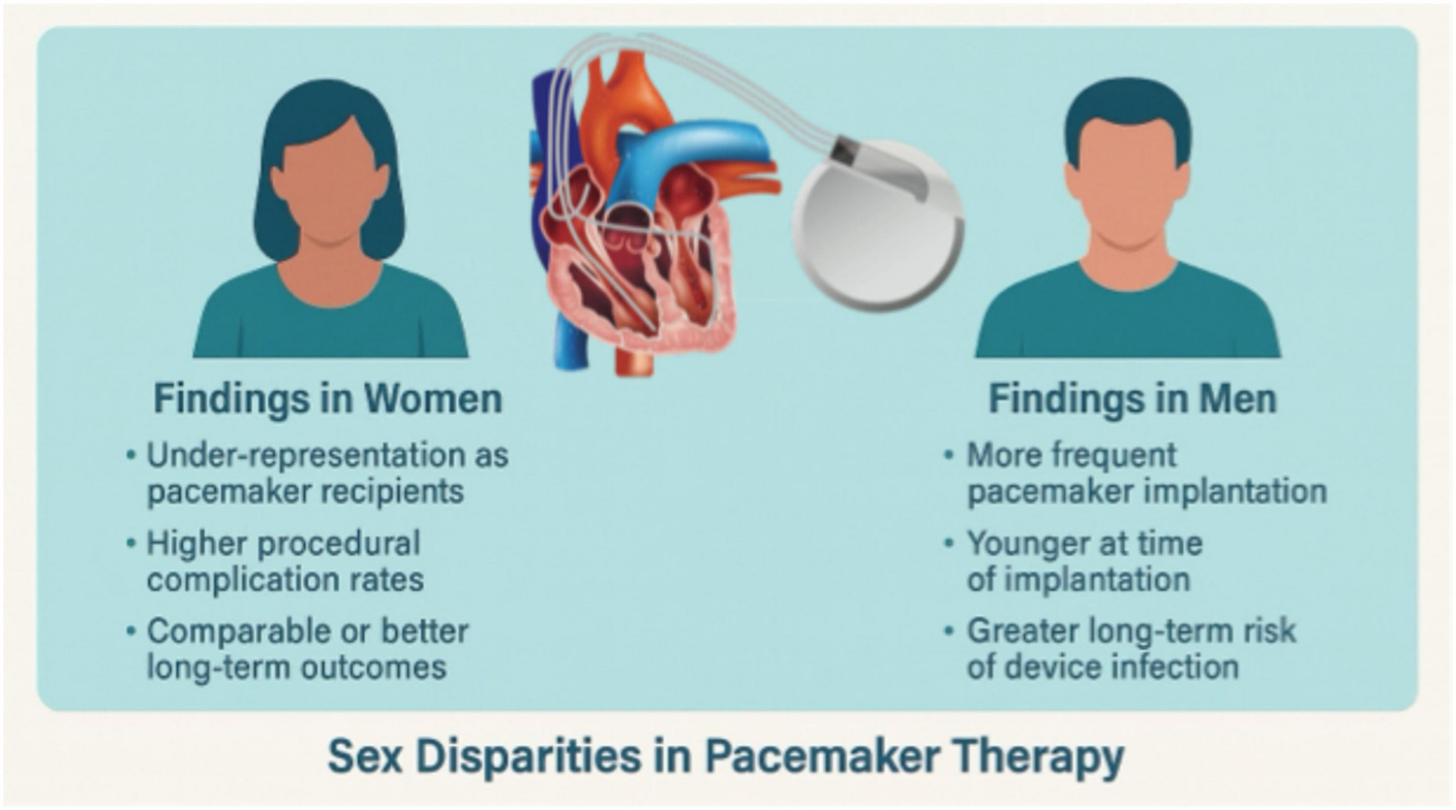
Graphical abstract summarising sex-based disparities in pacemaker therapy. The illustration compares key clinical findings in women (left) and men (right) across the pacemaker treatment pathway. Women are under-represented among recipients, experience higher procedural complication rates (pneumothorax, lead perforation), but demonstrate comparable or better long-term outcomes. In contrast, men receive pacemakers more frequently and at younger ages but are at greater long-term risk of device-related infections. These visual highlights the need for sex-specific considerations in patient selection, procedural technique, and long-term management to ensure equitable and optimised pacemaker care.

Finally, the interface of pacemaker therapy with other conditions unique to or more common in women (such as breast cancer needing radiation, how does pacemaker location affect treatment? or pregnancy, how to manage pacemakers in childbearing women?) is a niche but important topic. As more young women receive pacemakers for congenital or genetic bradycardias, issues like safe pacemaker management during pregnancy will require clear guidance (existing data are limited and not sex-comparative since men don't undergo pregnancy, but guidelines can be refined with more reports). Future research should aim to close the knowledge gaps by deliberately examining sex differences in pacemaker therapy at every step, from the molecular mechanisms of node degeneration to technical improvements to psychosocial outcomes. As one commentary noted, it is time to move beyond one-size-fits-all and ensure that evidence-based insights inform sex-specific best practices in device therapy. This will ultimately improve care for both women and men by acknowledging and addressing their distinct needs. Figure 1 and Table 1 summarise sex differences in pacemaker procedural disparities along with the corresponding evidence. Future studies should prioritise balanced recruitment, prespecified sex-stratified analyses, and mechanistic investigation to clarify whether observed differences reflect biological variation, healthcare delivery factors, or both.

**Table 1:** Summary of key sex-based differences in pacemaker therapy across the clinical pathway.

Domain	Findings in women	Findings in men	Key evidence
Epidemiology and utilisation	Lower overall implantation rates; receive pacemakers at older ages; under-representation in device datasets	Higher implantation rates; implanted at younger ages	Vijayarajan et al., 2022; Chen et al., 2018 (1, 3)
Indications for implantation	More sinus node dysfunction and sick sinus syndrome; fewer cases of AV block	More AV block and His–Purkinje disease	Chen et al., 2018; Yang et al., 2025 (3, 4)
Referral pathways	More emergency or urgent admissions; possible under-recognition of symptoms before severe presentation	More elective referrals	Vijayarajan et al., 2022 (1)
Device selection	Historically received fewer dual-chamber devices, but contemporary data show equal or higher dual-chamber use driven by sinus node dysfunction	More single-chamber ventricular devices, often due to AF	Nowak et al., 2010; Chen et al., 2018 (3, 10)
Procedural characteristics	Smaller venous anatomy; higher atrial pacing thresholds; lower P-wave amplitudes; more device visibility or pocket discomfort	Larger vasculature; lower thresholds; fewer cosmetic pocket concerns	Nowak et al., 2010 (10)
Acute complications	Higher risk of pneumothorax, pocket hematoma, and lead perforation; higher overall in-hospital complication rates	Lower acute complication risk	Vijayarajan et al., 2022; Cano et al., 2017 (1, 23)
Lead-related long-term issues	Similar long-term lead durability; potential higher risk of venous occlusion; may tolerate RV pacing better with lower pacing-induced cardiomyopathy risk	More pacing-induced cardiomyopathy and CRT upgrades	Riesenhuber et al., 2020; Rorsman et al., 2023 (2, 44)
Infection risk	Lower incidence but higher mortality when infection occurs	Higher incidence of device infection	Birnie et al., 2019; Sohail et al., 2013 (36, 39)
Tricuspid valve effects	Potentially greater haemodynamic impact of lead-associated tricuspid regurgitation	Similar risk of TR but better tolerated	Riesenhuber et al., 2020 (2)
Quality of life	Greater concerns regarding device visibility; lower physical function scores in some studies; benefit strongly from symptom relief	Higher physical function measures; similar symptom improvement	Barros et al., 2014 (46)
Leadless pacemakers	Underrepresented among recipients yet outcomes equivalent; slightly higher impedance but no clinical impact	Majority of recipients; comparable efficacy and safety	Mitacchione et al., 2023; Huang et al., 2024 (48, 49)
Conduction system pacing	Possibly greater benefit in CRT indications; strong response to LBBAP	Benefit present but less pronounced	Subzposh et al., 2024; Wu et al., 2024 (51, 52)

AV block, atrioventricular block; AF, atrial fibrillation; RV, right ventricular; CRT, cardiac resynchronisation therapy; TR, tricuspid regurgitation; LBBAP, left bundle branch area pacing; CIED, cardiac implantable electronic device.

## Conclusion

Sex disparities in pacemaker therapy are increasingly recognised, from who gets a pacemaker to the device type and outcomes. Women, despite developing bradyarrhythmias at older ages and from different causes, are under-referred for pacing and face higher procedural risks. They often require dual-chamber systems but, historically, have received fewer advanced pacing modes, though this gap is narrowing. Men, more frequently affected by AV block and receiving devices at a younger age, are at risk for long-term issues like cardiomyopathy and infections. Women experience higher rates of acute complications such as pneumothorax and hematomas, highlighting the need for refined techniques and preventive measures. However, women tend to have comparable or better long-term survival, likely due to fewer comorbidities and greater symptom relief. Both sexes benefit from improved quality of life, though men report higher physical function, and women may need more support with device acceptance.

New pacing technologies, like leadless pacemakers, eliminate leads and pockets, reducing complications, especially in women, and show similar success rates across sexes. Conduction system pacing can prevent dyssynchrony and heart failure, offering superior resynchronisation, particularly beneficial for women. Embracing these innovations enables personalised treatment based on anatomical and risk factors.

Recognising sex-specific differences highlights challenges and opportunities. Ensuring equitable referral, optimising implantation techniques, and tailoring follow-up are vital. However, the current evidence base is largely derived from high-income countries and registry-based studies, with limited representation from low and middle-income regions, which may limit the generalisability of these findings across different healthcare systems and resource settings. The goal is to deliver the right device at the right time, with appropriate management to maximise benefits and minimise complications. Research focused on sex disparities will foster more personalised, fair pacemaker care, improving outcomes for all bradycardia patients.
